# Characteristics and Phylogenetic Analysis of the Complete Chloroplast Genome of *Hibiscus sabdariffa* L.

**DOI:** 10.3390/ijms262211001

**Published:** 2025-11-13

**Authors:** Junyuan Dong, Qingqing Ji, Xingcai An, Xiahong Luo, Changli Chen, Tingting Liu, Lina Zou, Shaocui Li, Guanghui Du, Jikang Chen, Xia An

**Affiliations:** 1 Zhejiang Xiaoshan Institute of Cotton & Bast Fiber Crops, Zhejiang Institute of Landscape Plants and Flowers, Zhejiang Academy of Agricultural Sciences, Hangzhou 311251, China; 13964552682@163.com (J.D.); jiqingqing1001@163.com (Q.J.); xcan2001str@163.com (X.A.); luoxh@zaas.ac.cn (X.L.); chenchangli@zaas.ac.cn (C.C.); liutt@zaas.ac.cn (T.L.); zoulina1991@yeah.net (L.Z.); lishaocui@zaas.ac.cn (S.L.); 2 School of Agriculture, Yunnan University, Kunming 650500, China; dgh2012@ynu.edu.cn; 3 Institute of Bast Fiber Crops, Chinese Academy of Agricultural Sciences/Key Laboratory of Bast Fiber Biology and Processing, Ministry of Agriculture and Rural Affairs, Changsha 410221, China

**Keywords:** *Hibiscus sabdariffa* L., chloroplast genome, phylogenetic analysis

## Abstract

Roselle (*Hibiscus sabdariffa* L.) is a plant rich in bioactive constituents, serving as a unique material for the food and beverage industry and therapeutic applications. Despite its significant utility, few studies have focused on the molecular breeding of the plant. Chloroplasts are organelles in plant cells with independent genetic information, making them ideal for investigating plant phylogeny and genetic evolution. In this study, the roselle breeding material ‘Zhe Xiao Luo 1’ was selected to assemble and analyze the entire chloroplast genome using the Illumina NovaSeq X Plus platform. The phylogenetic relationships between roselle and other species within Malvaceae family, particularly within the genus Hibiscus, were clarified. The results showed that the complete chloroplast genome of roselle was 162,428 bp in length, with nucleotide proportions of 31.14% (A), 18.73% (C), 18.01% (G), 32.12% (T), and 36.74% (GC). It exhibited a typical tetrad structure consisting of four segments: the large single copy (LSC) region (90,327 bp), the small single-copy (SSC) region (19,617 bp), and two inverted repeat sequences (IRa and IRb, each 26,242 bp). A total of 130 genes were identified, including 37 tRNA genes, 8 rRNA genes, and 85 mRNA genes, and no pseudogenes were detected. Phylogenetic analysis using 23 revealed a clear phylogenetic relationship between *H. sabdariffa* and *H. esculentus* (okra) among all tested species. Building on previous research, this study further explored the functional annotation of genes in the roselle chloroplast genome, as well as its codon preference, repetitive sequences, simple sequence repeats (SSR), Ka/Ks ratio, nucleotide diversity (pi) analysis, and boundary analysis. The complete gene sequences have been uploaded to the NCBI database (accession number PX363576). This study provides evidence for elucidating the phylogenetic relationships and taxonomic status of *H. sabdariffa*, laying a theoretical foundation for studies on molecular mechanism resolution and cultivar development.

## 1. Introduction

Roselle (*Hibiscus sabdariffa* L.) is an annual herbaceous plant belonging to the genus Hibiscus within the Malvaceae family. Plants of the Hibiscus genus are rich in chemical constituents such as terpenoids, flavonoids, and polyphenols, which exhibit a wide range of pharmacological activities, including anti-tumor, antibacterial, anti-inflammatory, and antioxidant effects, and thus have significant research and application value [1]. Calyxes, the main product of roselle, are used to extract various bioactive compounds such as hibiscus pigment, hibiscus saponins, and hibiscus polysaccharides, serving as crucial raw materials for food, beverage, and pharmaceutical production [2]. The hydrolysate of roselle seed protein is used to enhance the antioxidant activity of foods and regulate the structure of the gut microbiota [3]. Roselle is also widely used as a bast fiber crop in the textile industry [4]. Roselle possesses rich germplasm diversity with over 200 species of hibiscus plants found worldwide; however, due to the lack of analysis on its molecular genetic basis, current variety development relies on screening natural variations and conventional hybridization, resulting in a shortage of innovative germplasm derived from extensive genetic diversity [2,5,6].

With the rapid development of high-throughput sequencing and analysis technologies, plant chloroplast genome sequencing has played a pivotal role in plant resource conservation, resource authenticity verification, and genetic diversity assessment [7,8,9]. For example, Song et al. (2024) compared 12 water-lily plastomes, revealed substantial structural divergence, and robustly resolved backbone relationships within an early-diverging angiosperm lineage, illustrating the power of plastid phylogenomics [10]. This demonstrates the potential of in-depth comparative plastome analyses to uncover critical evolutionary and genomic information. This is attributed to the stable maternal inheritance, high copy number (ease to obtain), and abundance of species-specific markers of chloroplast genomes. This technology has been widely applied to more than 1500 plant and algal species, yielding significant scientific results. The complete chloroplast genome data of *H. sabdariffa* was first published in 2022, providing valuable insights into the genetic diversity of this species. However, previous research on this genome was limited to basic genomic characteristics and phylogenetic analysis, leaving vast amounts of information unexplored and failing to provide sufficient research materials.

Therefore, this study aimed to clarify the core characteristics of the complete chloroplast genome of the Roselle breeding material ‘Zhe Xiao Luo 1′ through Illumina high-throughput sequencing and bioinformatics techniques, including gene functional annotation, codon preference, repetitive sequences, SSR distribution, Ka/Ks selection effects. We also intended to elucidate the phylogenetic relationships between roselle and other Malvaceae species (particularly Hibiscus and Abelmoschu genera). The results will fill the research gap in in-depth analysis of Roselle chloroplast genomes and provide crucial references for the development of genetic resource and molecular breeding practices (e.g., screening of specific gene markers), taxonomic refinement of Malvaceae plants, and research on the evolutionary mechanisms of chloroplast genomes.

## 2. Results

### 2.1. Chloroplast Genome Structure of Hibiscus sabdariffa L.

**Figure 1:**
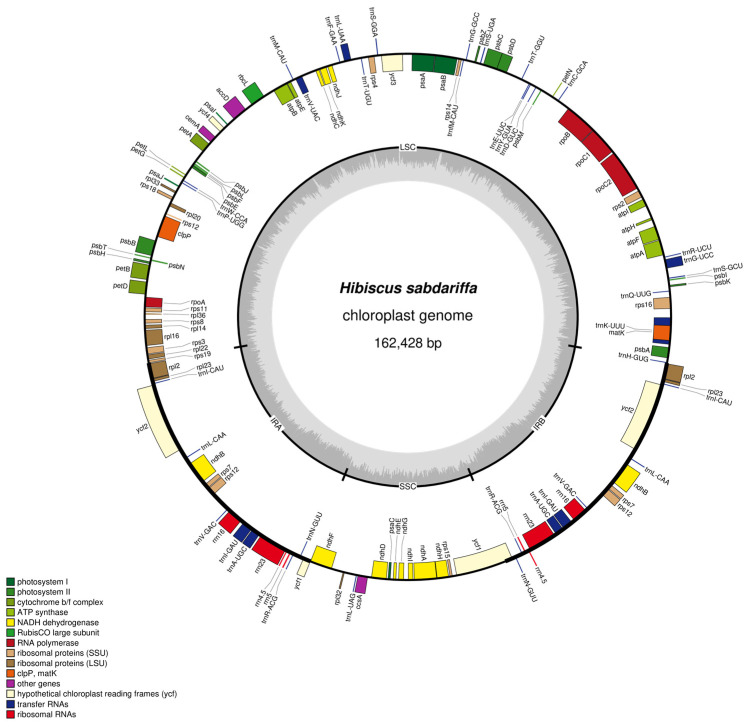
Map of *Hibiscus sabdariffa* L. chloroplast genome. Note: Genes with forward coding are located on the outer side of the circle, while those with reverse coding are on the inner side. The inner gray circle represents GC content.

The chloroplast genome of *Hibiscus sabdariffa* L. exhibits a classic tetrad structure comprising four segments: the Large Single Copy Region (LSC), with a sequence length of 90,327 bp; the small single-copy region (SSC), with a sequence length of 19,617 bp; the inverted repeat sequence a (IRa), with a sequence length of 26,242 bp; and the inverted repeat sequence b (IRb), with a sequence length of 26,242 bp (Figure 1, Table 1). The chloroplast genome of *Hibiscus sabdariffa* L. spans 162,428 bp. Nucleotide frequencies are as follows: A (31.14%), C (18.73%), G (18.01%), T (32.12%), and GC (36.74%). The GC content is relatively high.

The genome exhibits the highest GC content in large single-copy regions (LSC), reaching 34.46% across 31,130 base pairs; In small single-copy regions (SSC), T had the highest proportion at 34.97%, covering 6860 bp; In inverted repeat sequence a (IRa), GC had the highest proportion at 42.66%, covering 11,195 bp; In inverted repeat sequence b (IRb), GC had the highest proportion at 42.66%, covering 11,195 bp (Table 1).

### 2.2. Functional Annotation of Chloroplast Genes

Analysis of the chloroplast genome of *Hibiscus sabdariffa* L. identified a total of 130 genes, including 37 tRNA genes, 8 rRNA genes, 85 mRNA genes, and 0 pseudogenes (Table 2). These genes regulate photosynthesis and self-replication within the chloroplast, ensuring the proper execution of all vital functions. Additionally, five genes involved in other functions (such as mature enzymes and proteases) and four genes with unknown functions (ycf genes) were annotated.

A total of 44 genes were identified as influencing photosynthesis. Among the genes affecting chloroplast photosynthesis, 5 genes influence Photosystem I, and 15 genes influence Photosystem II. A total of 11 genes affect NADH dehydrogenase (with the ndhB gene duplicated twice); 6 genes affect the cytochrome complex; 6 genes affect ATP synthase; 1 gene affects 1,5-diphosphate carboxylase synthesis; and 0 genes affect photosynthetic pigment reductase (Table 2).

A total of 59 genes were identified as involved in chloroplast self-replication: 9 genes affecting large subunit ribosome synthesis, 12 genes affecting small subunit ribosome synthesis, 4 genes for RNA polymerase, 4 genes for tRNA synthesis, and 30 genes for rRNA synthesis (Table 2).

To ensure the accuracy of gene annotation verification, we compared the annotated genes of ‘Zhe Xiao Luo No. 1′ with those from the Korean test okra (MZ522720.1, sourced from NCBI). The results revealed high conservation: ‘Zhe Xiao Luo 1′ contained 130 genes (37 tRNAs, 8 rRNAs, 85 mRNAs), while the Korean test okra had 131 genes (37 tRNAs, 8 rRNAs, 85 mRNAs). The minor discrepancy of one mRNA may stem from variety-specific variation, consistent with the structural conservation observed in the chloroplast genomes of Malvaceae plants.

### 2.3. Codon Preference Analysis

A systematic analysis of codon usage characteristics in the chloroplast genome of *Hibiscus sabdariffa* L. revealed a total of 22,841 codons, with 22,763 codons involved in amino acid coding (excluding the stop codon Ter), encoding 19 amino acids. Terminator codons (Ter) exhibited three nucleotide compositions: UAA appeared in 43 codons with a preference of 1.6539; UAG appeared in 18 codons with a preference of 0.6924; UGA appeared in 17 codons with a preference of 0.6537 (Table 3).

Regarding codon preference, the UUA codon for leucine (Leu) exhibits the highest preference at 1.9836, occurring most frequently 794 times. Among all utilized codons, the AGC codon for serine (Ser) shows the lowest preference at 0.3588, appearing most often at 101 times. Regarding usage frequency, the AUU codon for isoleucine (Ile) was used most frequently at 979 times, with a preference of 1.4901. Excluding stop codons and non-coding codons, the UGC codon for cysteine (Cys) was used least frequently at 58 times, with a preference of 0.4604 (Table 3).

Methionine is exclusively initiated by the AUG codon (531 occurrences), with a codon preference value of 7—significantly higher than other codons—consistent with its uniqueness as the sole start codon. No other codons (such as AUA or AUC) are utilized, demonstrating the specificity of the start codon (Table 3).

**Figure 2:**
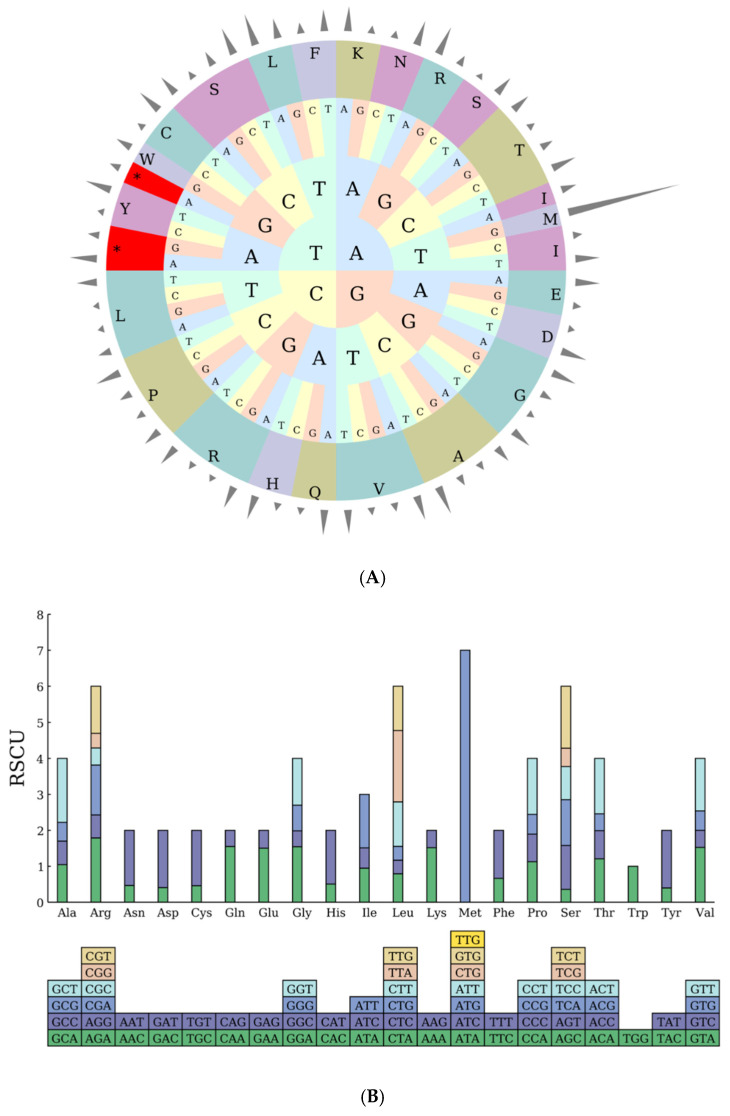
(**A**) RSCU Pie Chart. Note: The outermost cylinder represents the RSCU value, the middle layer consists of amino acids, and the innermost three layers represent codons. Different colors denote different amino acids (abbreviations are labeled on the outer ring, e.g., L for leucine, F for phenylalanine, etc.); the inner letters (A, T, C, G) indicate nucleotides; asterisks (*) mark codons with significant characteristics. (**B**) RSCU Histogram. Note: The squares below represent all codons encoding each amino acid, while the height of the columns above represents the total sum of RSCU values for all codons.

The RSCU pie chart generated from the above data (Figure 2A) visually presents the distribution pattern of amino acid-specific codons, facilitating the interpretation of codon usage preferences within the chloroplast genome of *Hibiscus sabdariffa* L.

### 2.4. Repeat Sequence Analysis

**Figure 3:**
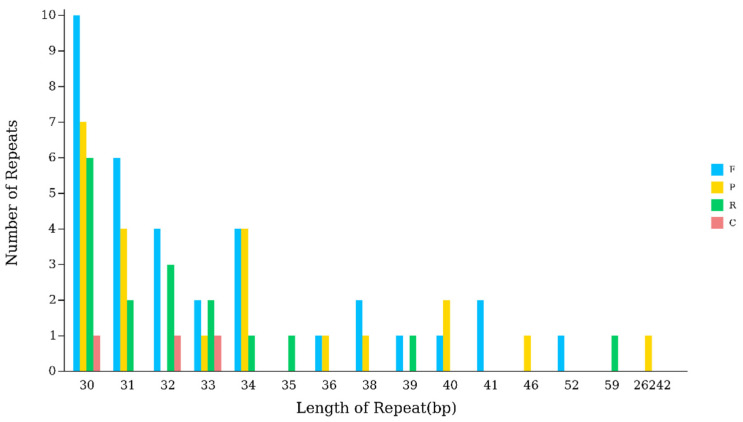
Analysis of scattered sequence repeats in the chloroplast genome of *Hibiscus sabdariffa* L. Note: The horizontal axis represents the length of scattered repetitive sequences, while the vertical axis represents the number of scattered repetitive sequences. F denotes forward repeats, P denotes palindromic repeats, R denotes reverse repeats, and C denotes complementary repeats.

Scattered repetitive sequences are non-contiguous segments of repetitive DNA distributed throughout the genome, primarily composed of transposable elements. The chloroplast genome of *Hibiscus sabdariffa* L. contains 34 forward repeats (F), 22 palindromic repeats (P), 17 reverse repeats (R), and 3 complementary repeats (C), totaling 76 scattered repeat sequences. The lengths of these scattered repeat sequences mostly fall within the 30–39 bp range, except for a single palindromic repeat reaching 26,242 bp. Among them, the 30 bp repeats are the most numerous, totaling 24 (Table 4) (Figure 3).

### 2.5. Simple Sequence Repeat (SSR) Analysis

Simple Sequence Repeats (SSR), also known as microsatellite DNA, refer to tandem repeat sequences composed of 1 to 6 nucleotides within genomes. They are widely distributed across the genomes of various eukaryotic organisms.

**Figure 4:**
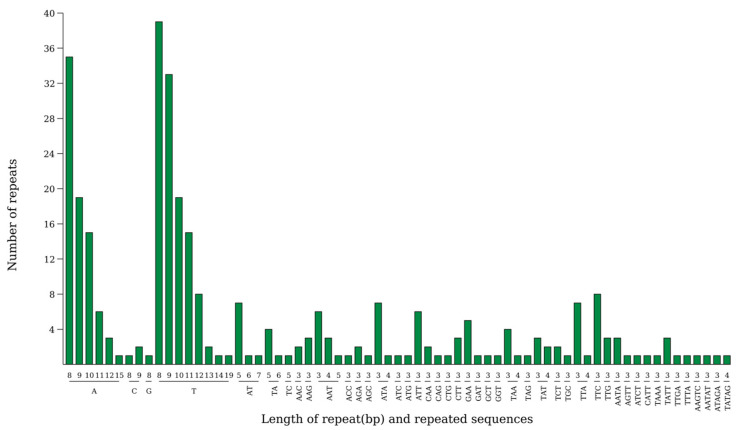
Analysis of simple sequence repeats in the chloroplast genome of *Hibiscus sabdariffa* L. Note: The horizontal axis represents SSR repeat units, and the vertical axis represents the number of repeat units.

Analysis revealed the presence of 315 SSRs in the chloroplast genome of *Hibiscus sabdariffa* L., comprising 225 in the large single-copy region (LSC), 44 in the small single-copy region (SSC), and 23 each in the two inverted repeat sequences (IRa and IRb). Among the five types of base repeats, single-base repeats occurred most frequently, totaling 201 instances; followed by triplet repeats, which appeared 85 times; other base repeats included 15 instances of diplet repeats, 12 instances of quadruplet repeats, and 4 instances of quintuplet repeats. Regarding SSR repeat unit types, A and T repeats were significantly more frequent than other types (Figure 4).

### 2.6. Ka/Ks Analysis

Through advanced analysis, when using *Hibiscus sabdariffa* L. as the reference, the average Ka/Ks ratios of genes from the remaining 8 test species ranged between 0.17 and 0.3: the highest value (0.3054) was detected in *Abelmoschus sagittifolius* (NC_053354), while the lowest (0.1737) was observed in *Hibiscus sinosyriacus* (MZ367751). These results align with the phylogenetic divergence levels among the species, and also provide a foundational reference for the subsequent analysis of nucleotide diversity.

**Figure 5:**
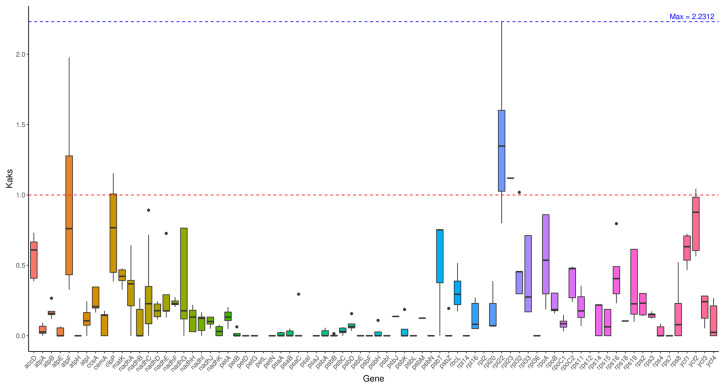
Ka/Ks analysis. Note: The horizontal axis represents gene names, while the vertical axis denotes Ka/Ks ratios. In the box plot, the upper and lower endpoints of the vertical lines above and below the rectangle indicate the upper and lower bounds of the data, respectively. The thick line within the rectangle represents the median, while the upper and lower edges of the rectangle denote the upper and lower quartiles. Data points extending beyond the upper and lower bounds of the rectangle are considered outliers.

Analysis was conducted on individual genes. Most genes exhibit Ka/Ks < 1, indicating purification selection and functional conservation. Among these, certain genes show Ka/Ks = 0 when compared to *Hibiscus sabdariffa* L., demonstrating exceptionally high conservation. Certain genes, such as the atpF gene in *Abelmoschus moschatus* (NC_053355) and *Abelmoschus sagittifolius* (NC_053354), exhibit Ka/Ks ratios >1 (1.9765), indicating positive selection pressure (Figure 5).

### 2.7. Nucleic Acid Diversity Pi Analysis

**Figure 6:**
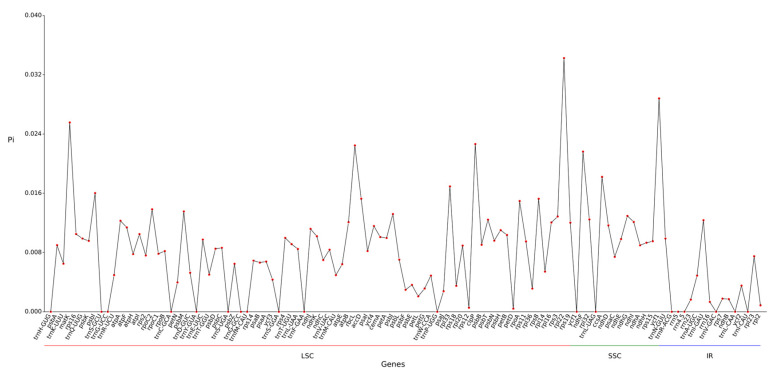
Line chart of gene Pi value.

Analysis of nucleotide diversity in the chloroplast genome of *Hibiscus sabdariffa* L. revealed an average nucleotide diversity (Pi) of 0.0079 across 113 gene regions (Figure 6). The maximum Pi value in the SSC region was 0.011628, while the Pi values in the LSC and IR regions were 0.008476 and 0.002836, respectively. The overall Pi values in the IR region were significantly lower, reflecting the sequence conservation in this region. Genes contained within the IR region (such as ribosomal RNA genes) typically perform fundamental and critical functions, are subject to strong purifying selection, and thus exhibit low mutation rates. A total of 6 highly variable regions (Pi ≥ 0.02) were identified: LSC’s *rpl22* (0.03425), *4.matK* (0.02555), *68.clpP* (0.02264), *49.rbcL* (0.02244); and *14.ycf1* (0.0288) and *2.ndhF* (0.02162) in SSC.

The Pi values in the LSC and SSC regions are relatively high and exhibit multiple fluctuation peaks, indicating that these two regions are major hotspots for genetic variation. This disparity may be related to the diversity of gene functions within these regions and the heterogeneity of selective pressures (Figure 6).

### 2.8. Boundary Analysis

Analysis of chloroplast genome boundaries in the remaining eight tested species of *Hibiscus sabdariffa* L. revealed that all these plants possess four distinct boundaries: JLB, JSB, JSA, and JLA. Key genes located near the IR boundary include *rpl2* and *ycf1* in these plants. The average size of the JLB region in the tested species of the Hibiscus genus is 89,822 bp, differing by 1672 bp compared to the 88,209 bp in the Okra genus. Within the IR region, *Hibiscus sabdariffa* L. and its genus Hibiscus are significantly smaller than those of the genus Acacia.

The JLB boundary is located within the region associated with the *rpl2* gene coding domain. Its position varies significantly across different plant chloroplast genomes. For example, the variation is markedly smaller in species of the same genus Hibiscus, such as *Hibiscus syriacus*, compared to species of the genus Abelmoschus, such as Abelmoschus esculentus.

The JSB boundary is located within the coding region of the ycf1 gene. In most plants, the majority of the ycf1 gene resides in the IRb, with only a small portion extending into the SSC. Within the Hibiscus genus, *Hibiscus sabdariffa* and Hibiscus rosa-sinensis exhibit significantly smaller extensions compared to the other two species of the genus.

The JSA boundary is also associated with the coding region of the *ycf1* gene, with part of the *ycf1* gene sequence located in IRa and another part in SSC. In different plants, the lengths of the *ycf1* gene fragments in IRa and SSC vary, with *Hibiscus sabdariffa* showing significant differences from other tested species. The evolutionary diversity reflected in the “cross-region distribution” of the *ycf1* gene across the IR/SSC boundary among species can be utilized for phylogenetic analysis.

**Figure 7:**
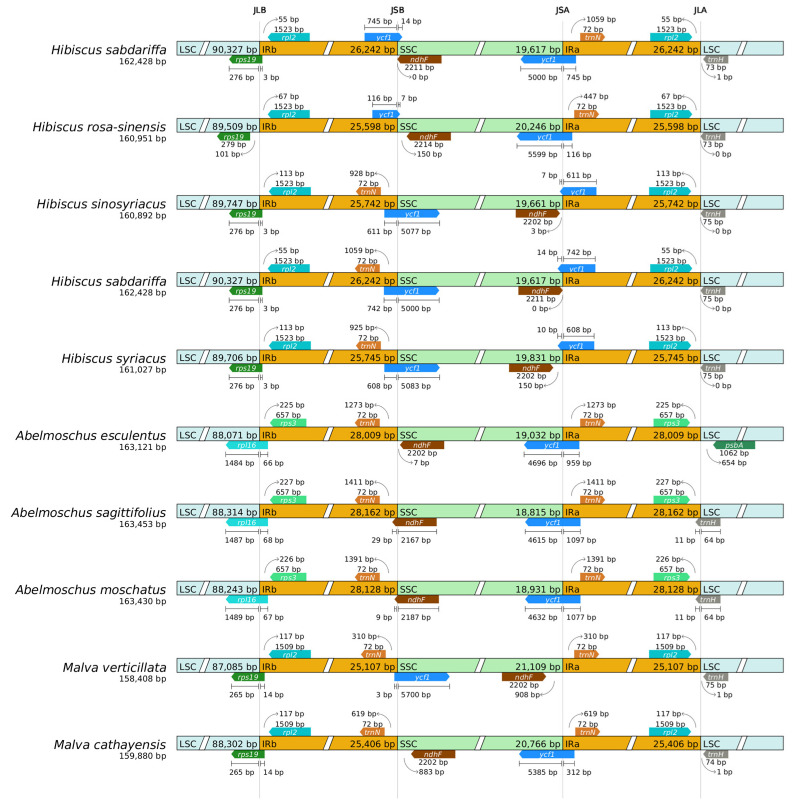
IR/SC boundary analysis.

The primary *trnH* gene at the JLA boundary is located in the LSC region, exhibiting minimal variation within the same genus but significant differences across genera. Taking the Hibiscus genus as an example, the variation is extremely minor among *Hibiscus sabdariffa*, *Hibiscus sinosyriacus*, *Hibiscus syriacus*, and *Hibiscus rosa-sinensis*, with only *Hibiscus sabdariffa* being 1 bp away from the JLA boundary (Figure 7).

### 2.9. Phylogenetic Analysis

**Figure 8:**
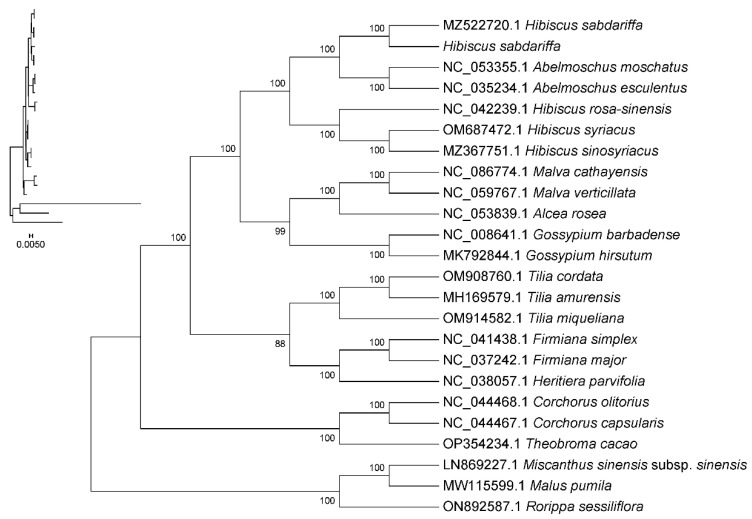
Phylogenetic tree constructed based on chloroplast genome sequences. Note: (1) Sequence names correspond to species Latin names. (2) Branch length: Also known as genetic variation or evolutionary distance. Represents the degree of change in evolutionary branches; shorter lengths indicate smaller differences and closer evolutionary distances. (3) Distance scale: The unit length for measuring differences between organisms or sequences, equivalent to the scale of an evolutionary tree. (4) Self-expansion value: Used to display the reliability of evolutionary tree branches. Typically represented by a number between 0 and 100.

By employing shared CDS for phylogenetic analysis, a systematic evolutionary tree was constructed among *Hibiscus sabdariffa* L., three other species within the genus Hibiscus, 16 species within the family Malvaceae Juss., and three outgroup species. This study explores the phylogenetic relationships of *Hibiscus sabdariffa* L. The results indicate that *Hibiscus sabdariffa* is phylogenetically more distant from the three selected species of the same genus—*Hibiscus sinosyriacus*, *Hibiscus syriacus*, and *Hibiscus rosa-sinensis*—suggesting the complex composition of the Hibiscus genus. *Abelmoschus esculentus* and *Abelmoschus moschatus* of the genus Abelmoschus are most closely related to the genus Hibiscus; next are *Malva cathayensis* and *Malva verticillata* of the genus Malva; followed by *Gossypium hirsutum* and *Gossypium barbadense* of the genus *Gossypium* Linn. *Tilia cordata*, *Tilia miqueliana*, and *Tilia amurensis* of the genus Tilia L. are distantly related; *Malus pumila*, *Arabidopsis thaliana*, and *Oryza sativa* are the most distantly related outgroups (Figure 8).

## 3. Discussion

### 3.1. Comparison of Chloroplast Genome Structure with Existing Study

As a crop offering multi-purpose applications with economic importance, a developing genetic diversity was detected according to previous studies [2,5,11]. This phenomenon was largely attributed to the rapid development of cross-regional introduction and hybrid breeding [12]. According to the main direction of industrialization, roselle was selected for edible products, ornamental uses, and dual-purpose breed. The previous research revealed the complete chloroplast genome of ornamental species [13]. It is the first time that the complete chloroplast genome of dual-purpose roselle was released.

Roselle has a long history of culinary use and offers health benefits such as antioxidant and anti-cancer properties [14]. The hibiscus flower contains abundant water-soluble pigments (like anthocyanins) that give it a yellow or pale yellow hue with rose or chestnut undertones [15]. The tested ‘Zhe Xiao Luo 1′ hibiscus cultivar, suitable for both culinary and ornamental purposes, achieves value enhancement while retaining the inherent advantages of roselle: Its calyx exhibits a vivid purplish-red hue with exceptional ornamental appeal. Moreover, the accumulation of phenolic acids, flavonoids, and anthocyanins, along with organic acids like citric acid and hydroxycitric acid within the plant, not only enriches its nutritional profile but also provides a robust material foundation for advanced applications such as natural pigment extraction and functional food development.

While the initial report by Kwon, S.-H. et al. (2022) was primarily focused on the assembly and basic annotation of the ornamental variety’s chloroplast genome, our research delves deeper into functional annotation, codon usage bias, repetitive sequences, SSRs, selective pressure (Ka/Ks), nucleotide diversity (Pi), and IR boundary dynamics [13]. This multifaceted approach not only confirms the structural conservation of the chloroplast genome within *Hibiscus sabdariffa* but also unveils the evolutionary forces shaping its genome and identifies potential molecular markers for future phylogenetic and population genetics studies.

Kwon, S.-H. et al. [13] found that the large single-copy region (LSC), small single-copy region (SSC), and two inverted repeat sequences measured 90,327 bp, 19,617 bp, and 26,242 bp, respectively. These data correspond to the sequence lengths measured in this study: the large single copy region (LSC) at 90,327 bp, the small single copy region (SSC) at 19,617 bp, and the inverted repeat sequence (IR) at 26,242 bp. This reflects intraspecific consistency. Among these, A, C, G, T, and GC account for 31.14%, 18.73%, 18.01%, 32.12%, and 36.74% of the total, respectively. The GC content aligns with the general average level of 36.72% observed in Malva species [16].

### 3.2. Conservation and Difference Analysis of Chloroplast Gene Functional Annotation in Hibiscus sabdariffa

In the functional testing of chloroplast genes, a total of 130 genes were identified, including 37 tRNA genes, 8 rRNA genes, 85 mRNA genes, and 0 pseudogenes. The experimental results for tRNA and rRNA genes align with those reported by Kwon, S.-H. et al. [13]. However, for mRNA genes, this study identified fewer than the 86 genes previously determined by their research. Although the structure and function of chloroplast genomes are generally considered conserved, a large number of genetic variations have been discovered in the chloroplasts of *Hibiscus sabdariffa* [17]. These genetic variations can be utilized to develop molecular markers for identifying *Hibiscus sabdariffa* and distinguishing different varieties [18].

A systematic analysis of codon usage characteristics in the chloroplast genome of *Hibiscus sabdariffa* L. revealed a total of 22,841 codons, with 22,763 codons involved in amino acid coding (excluding the stop codon Ter), encoding 19 distinct amino acids. Terminator codons (Ter) consist of three nucleotide sequences: UAA occurs in 43 codons with a preference of 1.6539; UAG occurs in 18 codons with a preference of 0.6924; UGA occurs in 17 codons with a preference of 0.6537.

### 3.3. Evolutionary Significance of Repetitive Sequences and SSRs in the Hibiscus sabdariffa Chloroplast Genome

Repetitive motifs are widely distributed throughout the chloroplast genome and play a significant role in genomic evolution [19,20]. Analysis of scattered repeats and simple sequence repeats revealed four types of oligonucleotide repeats in the chloroplast genome of *Hibiscus sabdariffa* L.: 34 forward repeats (F), 22 palindromic repeats (P), 17 reverse repeats (R), and 3 complementary repeats (C), totaling 76 scattered repeats. This pattern is consistent with other representative species within the same genus [21]. The lengths of scattered repetitive sequences mostly fall within the 30–39 bp range, except for a single palindromic repeat reaching 26,242 bp. The most abundant length is 30 bp, occurring 24 times, while the least frequent length is 26,242 bp, occurring only once. Forward-oriented repetitive sequences are the most numerous, totaling 34, whereas complementary repeats are the least common, numbering only 3. *Hibiscus sabdariffa* L. The chloroplast genome contains a total of 315 SSRs, including 225 in the large single-copy region (LSC), 44 in the small single-copy region (SSC), and 23 each in the two inverted repeat sequences (IRa and IRb). Their distribution pattern is similar to that observed in other representative species of the Malvaceae family, such as *Hibiscus syriacus* and *Hibiscus rosa-sinensis* [21]. SSR analysis of the current study indicates that the majority of mononucleotide SSRs (A/T motifs) and dinucleotide SSRs (AT/TA motifs) are significantly more abundant than other nucleotide repeat patterns. These findings are supported by previous studies on the chloroplast genome of Hibiscus [22].

### 3.4. Ka/Ks Ratio and Nucleotide Diversity: Insights into Selection Pressure and Variation Hotspots

The non-synonymous (KA) and synonymous (KS) patterns of nucleotide substitution are nucleotide substitution patterns that serve as key indicators of biological evolution, with the Ka/Ks ratio used to reflect selective pressure on genes [23]. Following advanced analysis, when compared to *Hibiscus sabdariffa* L., the Ka/Ks ratios of genes from the remaining eight test species averaged between 0.17 and 0.3. The highest value was observed in *Abelmoschus sagittifolius* (NC_053354) at 0.3054. The lowest value was observed in *Hibiscus sinosyriacus* (MZ367751) at 0.1737. These results align with their phylogenetic divergence levels.

Analysis of nucleotide diversity in the chloroplast genome of *Hibiscus sabdariffa* L. revealed an average Pi value of 0.0079 across 113 gene regions. Among these, the SSC region exhibited the highest average nucleotide diversity at 0.011628, while the LSC region showed a Pi of 0.008476, and the IR region recorded a nucleotide diversity of 0.002836. Its nucleic acid diversity falls within the range of Hibiscus nucleic acid diversity (0.001–0.0933), while also reflecting the relatively high degree of variation across different regions of the Hibiscus chloroplast genome [21].

### 3.5. Boundary Dynamics of Chloroplast Genomes and Their Taxonomic Implications

In boundary analysis, significant positional variations are observed across chloroplast genomes of different plant species. For instance, the fluctuations in species within the *Hibiscus genus*, such as *Hibiscus rosa-sinensis*, are markedly smaller than those in species like *Abelmoschus esculentus* of the Abelmoschus genus. The chloroplast genomes of these plants all possess four boundaries: JLB, JSB, JSA, and JLA. Key genes located near the IR boundary include *rpl2* and *ycf1* in these plants. The average size of the JLB region in the tested species of the genus Hibiscus was 89,822 bp, compared to 88,209 bp in the genus Abelmoschus, a difference of 1672 bp. This value is slightly larger than the range of 1079–1469 bp reported by Kwon S.-H. et al. (2023) [16], which is likely due to sampling error resulting from insufficient test samples.

Within the IR region, *Hibiscus sabdariffa* L. and its genus Hibiscus are significantly smaller than the genus Abelmoschus, likely due to differences in the *rps19* and *rps16* genes. The dynamic changes observed at IR boundaries offer significant insights into evolutionary patterns at the population genetics and phylogenetic levels [22]. Closely related species typically exhibit high similarity at chloroplast genome junctions compared to distantly related species, consistent with our findings. Comparing the evolutionary diversity of IR segment boundaries among Malvaceae species can effectively serve as a tool for phylogenetic inference [22].

In most plants, the majority of the *ycf1* gene resides within the IRb region, with only a small portion extending into the SSC. Within the genus Hibiscus, the extensions observed in *Hibiscus sabdariffa* and *Hibiscus rosa-sinensis* are significantly smaller compared to the other two *Hibiscus species*.

### 3.6. Evolutionary and Functional Implications of the ycf1 Gene

Our boundary analysis revealed that the *ycf1* gene exhibits a distinctive pattern of spanning the IR/SSC junctions in *Hibiscus sabdariffa* L., a feature that may indicate lineage-specific rearrangements during plastome evolution within the Malvaceae family. This structural characteristic of *ycf1* warrants deeper discussion given its pivotal role in chloroplast biology. Functional genetic studies have unequivocally demonstrated that *ycf1* is an essential gene in higher plants, as its knockout results in lethality, underscoring its indispensable, non-redundant function in chloroplast biogenesis or maintenance [24]. This places *ycf1* among a small subset of plastid-encoded genes whose function cannot be compensated by the nuclear genome.

Intriguingly, despite its critical function, the *ycf1* protein displays a unique evolutionary trajectory. Only its N-terminal domain exhibits clear conservation across streptophytes spanning over 700 million years of evolution, while the C-terminal domain contains a motif that has been conserved for over 500 million years [25]. The IR/SSC boundary shifts we observed, which directly affect the fragmentation and distribution of the *ycf1* coding sequence, could therefore reflect evolutionary experiments in balancing the structural and functional constraints of this essential gene with the dynamic nature of plastome reorganization.

Furthermore, the *ycf1* locus exemplifies the intricate interplay between plastid and nuclear genomes—a defining feature of streptophyte evolution that was crucial for the emergence of land plants [26]. The essential nature of the plastid-encoded *ycf1* necessitates precise coordination with nuclear-encoded factors. The boundary variations we identified among Hibiscus and related genera provide a natural experiment to investigate how the nuclear genome responds to changes in the structure and localization of an essential plastid gene. Expanding comparative datasets across diverse streptophyte lineages will be crucial in elucidating how such plastid variations correlate with nuclear regulatory responses, ultimately helping to identify conserved mechanisms governing organelle–nucleus coevolution. Thus, the *ycf1* gene in *H. sabdariffa* serves not only as a marker for phylogenetic and boundary analyses but also as a promising model for probing the fundamental principles of inter-compartmental coordination in plant cells.

### 3.7. Phylogenetic Position of Hibiscus sabdariffa and Evolutionary Relationships Within Malvaceae

The protein-coding regions and conserved sequences of the chloroplast genome can be utilized for phylogenetic analysis and studies on variety evolution [27]. By constructing a phylogenetic tree, *Hibiscus sabdariffa* was found to be distantly related to the three selected species of the same genus *Hibiscus sinosyriacus*, *Hibiscus syriacus*, and *Hibiscus rosa-sinensis*. This indicates the complex composition of species within the Hibiscus genus. In comparative analyses with other genera, species of the genus Hibiscus show close phylogenetic relationships with those of the genus Malvaceae. Numerous prior studies have demonstrated this conclusion [28,29].

Building upon this research, further studies could include: determining the chloroplast genomes of other *Hibiscus sabdariffa* L. cultivars to enrich the *Hibiscus sabdariffa* L. gene bank; identifying specific loci to conduct gene marker-related research; and comparing and integrating data from other crops within the same family or genus to study and analyze evolutionary relationships.

## 4. Materials and Methods

### 4.1. Materials and Sequences

The test roselle sample for sequencing was from the breeding material ‘Zhe Xiao Luo No. 1’ of *Hibiscus sabdariffa* L., which was cultivated in field plots at the Zhejiang Institute of Landscape Plants and Flowers (Zhejiang Xiaoshan Cotton and Hemp Research Institute) (30°07′ N, 120°23′ E). During sampling, mature leaves were selected from the same healthy, disease-free plant. The leaves were washed with ultrapure water to remove impurities and blotted dry with absorbent paper to ensure no residue remains. Then, the samples were quick-frozen in liquid nitrogen and placed in an EP tube pre-chilled with dry ice for 10 min. After removal, the samples were preserved with dry ice for subsequent experiments.

Total DNA of ‘Zhe Xiao Luo No.1’ was extracted using a universal plant DNA extraction kit (Jisihuiyuan D312, Nanjing Jisihuiyuan Biotechnology Co., Ltd., Nanjing, China) and subjected to paired-end (PE) sequencing on the Illumina NovaSeq X Plus platform (Illumina, Inc., San Diego, CA, USA).

### 4.2. Genome Assembly and Result Control

SPAdes2 (v3.10.1) [30] software for assembling the core modules of the chloroplast genome, with k-mer values set to 55, 87, and 121, respectively. Assembly was performed without a reference genome. To address scenarios where complete circular genome sequences cannot be directly obtained in a single pass due to characteristics of second-generation sequencing, genomic repetitive sequences, or genome-specific structures, additional strategies were employed to obtain complete circular genome sequences.

The assembly of the core module of the chloroplast genome follows the following steps:

Contig assembly: Use SPAdes2 (v3.10.1) software to assemble chloroplast DNA (cpDNA) sequences, yielding the SEED sequence of the chloroplast genome. Extend the aforementioned SEED sequence via kmer iterative extension. If the extension yields a single contig, this contig is directly designated as the pseudo-genome sequence, and the process proceeds to the pseudo-genome sequence correction step for subsequent analysis. If no single contig is obtained, the SSPACE (v2.0) software [31] is employed to assemble the contig sequences from the previous step, constructing scaffold sequences.

Gap filling: Use the Gapfiller software (v2.1.1) [32] to fill gaps in the scaffold sequences. If gaps remain unfilled after completion, design species-specific primers, perform PCR amplification and sequencing validation, then reassemble the sequencing results until a complete pseudo-genome sequence is obtained.

Sequence correction and confirmation: Align the pseudo-genome sequences obtained from raw sequencing data. Based on the alignment results, correct the pseudo-genome sequences to enhance sequence accuracy. Based on the typical structural features of chloroplast genomes, the corrected pseudo-genome sequence was subjected to coordinate realignment, ultimately yielding the complete chloroplast genome sequence. Due to the conserved and rearranged nature of chloroplast genomes, three aspects of quality control are implemented for assembled chloroplast genomes to ensure the accuracy of testing results: by comparing the genome, metrics such as genome coverage and insert size are calculated; using the reference sequence for genome alignment, analyses of conserved regions, rearrangements, and other collinearity features are performed; structural information from the reference sequence is utilized to compare differences between the two genomes.

### 4.3. Comprehensive Data Analysis

#### 4.3.1. Codon Preference Analysis

Due to codon degeneracy, each amino acid corresponds to at least one codon and at most six codons. The usage rates of codons in genomes vary significantly across different species and organisms. This inequality in the utilization of synonymous codons is termed codon preference (Relative Synonymous Codon Usage, RSCU). This preference is considered a combined result of natural selection, species mutations, and genetic drift.

The calculation method is as follows: (Number of codons encoding a specific amino acid/Total number of codons encoding that amino acid)/(1/Number of codon types encoding that amino acid), and (Actual usage frequency of a codon/Theoretical usage frequency of that codon). Calculations performed using a custom Perl script.

#### 4.3.2. Repetitive Sequence Analysis

Scattered repetitive sequences represent a distinct type of repetitive sequence from tandem repeats, exhibiting a dispersed distribution across the genome. Repetitive sequences were identified using the vmatch (v2.3.0) [32] software in conjunction with a Perl script. Its parameters are set as follows: minimum length = 30 bp, Hamming distance = 3. Four identification modes are available: forward, palindromic, reverse, and complement.

#### 4.3.3. Simple Sequence Repeat (SSR) Analysis

SSR (Simple Sequence Repeats) markers are tandem repeat sequences composed of several nucleotides (typically 1 to 6 nucleotides) as the repeating unit, extending to dozens of nucleotides in length. SSR markers on the chloroplast genome are termed cpSSR markers. We employed the MISA (Micro Satellite Identification Tool, v1.0) [33] software for cpSSR analysis, with parameters set as follows: 1–8 (single-base repeats occurring 8 times or more), 2–5, 3–3, 4–3, 5–3, 6–3.

#### 4.3.4. Ka/Ks Ratio Analysis

If a base mutation results in an amino acid change, it is termed a non-synonymous mutation, and its mutation rate is referred to as the non-synonymous mutation rate (Ka). Conversely, a mutation that does not alter the amino acid is termed a synonymous mutation, and its mutation rate is referred to as the synonymous mutation rate (Ks). Non-synonymous mutations are generally subject to natural selection. The ratio of non-synonymous mutation rate (Ka) to synonymous mutation rate (Ks) indicates the type of selective pressure acting on the sequence. A ratio greater than 1 indicates positive selection effects, while a ratio less than 1 indicates purifying selection. Select plants *Hibiscus sabdariffa*, *Hibiscus sinosyriacus*, *Hibiscus syriacus*, *Hibiscus rosa-sinensis*, *Abelmoschus esculentus*, *Abelmoschus moschatus*, *Abelmoschus sagittifolius*, *Malva cathayensis*, *Malva verticillata* for advanced analysis, totaling 9 plant species. Perform multiple sequence alignment of gene sequences using the MAFFT (v7.427, auto mode) [34] software. Calculate the Ka/Ks ratio of the genes using the Ka/Ks_Calculator (v2.0) [35] software, with the following parameter settings: -mMLWL–c11 (11-Bacterial and Plant Plastid Code).

#### 4.3.5. Nucleotide Diversity (π) Analysis

Nucleic acid diversity (pi) reveals the extent of variation in nucleic acid sequences across different species. Regions exhibiting higher variability may serve as potential molecular markers for population genetics. Perform global alignment of homologous gene sequences across different species using the MAFFT software (v7.427, auto mode). Calculate the PI value for each gene using DNASP5 v5.10.1 [36]. To visualize boundary information, further processing is conducted using the CPJSdraw cloud platform tool from Nanjing Jisihuiyuan Biotechnology Co., Ltd. This experiment employed the default parameters of the CGVIEW software [13] to conduct comparative analyses of chloroplast genome structures among closely related species. During genomic evolution, IR boundaries undergo expansion and contraction, causing certain genes to enter IR regions or single-copy regions. A script was written using Perl’s SVG module to visualize boundary information. Perform genome alignment using Mauve (v2.3.1) [37] with default parameters to assess homology and synteny among chloroplast sequences.

### 4.4. Chloroplast Genome Annotation and Genetic Map Construction

#### 4.4.1. Raw Data Filtering

This experiment utilized the fastp (v0.20.0) [38] software to filter the raw data. During filtering, sequencing adapters and primer sequences within the reads were trimmed, and reads with an average quality score below Q5 or containing more than five Ns were excluded.

#### 4.4.2. Chloroplast Genome Annotation

To ensure the accuracy of the annotated complete chloroplast genome, two methods were employed to annotate the chloroplast genome. First, use Prodigal (v2.6.3) [39] to annotate chloroplast coding sequences (CDS), employ HMMER (v3.1b2) [40] to predict rRNA, and utilize Aragorn (v1.2.38) [41] to predict tRNA. Second, based on closely related species already published on NCBI, their gene sequences were extracted. These sequences were then aligned and assembled using BLAST (v2.6) [42] to obtain a second annotation result. Subsequently, manually reviewing the differing genes between the two annotation sets allowed for the removal of erroneous annotations and redundant annotations, while confirming multi-exon boundaries to yield the final annotation.

#### 4.4.3. Chloroplast Genome Map Construction

After completing all preparatory work, we proceeded to construct the chloroplast genome map using the collected data. For this experiment, the OGDRAW [43] software was employed to generate the chloroplast genome map.

#### 4.4.4. Phylogenetic Tree Construction

To construct a phylogenetic tree, complete chloroplast genomes from 23 different species were selected from the NCBI database. *Malus pumila*, *Oryza sativa*, *Arabidopsis thaliana*, and other species were selected as outgroups. Plants belonging to the same genus and family as *Hibiscus sinosyriacus*, *Hibiscus syriacus*, *Hibiscus rosa-sinensis*, and *Hibiscus sabdariffa* were selected to investigate their phylogenetic relationships. The following 16 species belonging to *Malvaceae Juss* were simultaneously selected, with *Abelmoschus esculentus*, *Abelmoschus moschatus*, *Firmiana simplex*, *Firmiana major*, *Theobroma cacao*, *Gossypium hirsutum*, *Gossypium barbadense*, *Corchorus olitorius*, *Corchorus capsularis*, *Tilia cordata*, *Tilia miqueliana*, *Tilia amurensis*, *Alcea rosea*, *Heritiera parvifolia*, *Malva cathayensis*, *Malva verticillata* used to construct the phylogenetic tree.

This experiment employed whole-genome phylogenetic analysis. Circular sequences were aligned with identical starting points, and interspecies sequences were multi-sequence aligned using MAFFT software (v7.427, auto mode). The aligned data were trimmed with trimAl (v1.4.rev15), followed by construction of a maximum likelihood phylogenetic tree using RAxML (v8.2.10) [44] software with the GTRGAMMA model and rapid Bootstrap analysis (bootstrap = 1000) to construct a maximum likelihood phylogenetic tree.

## 5. Conclusions

The complete chloroplast genome of *Hibiscus sabdariffa* L. breeding line ‘Zhe Xiao Luo No.1′ was assembled, annotated and analyzed using high-throughput sequencing and bioinformatics tools, which provided key data for clarifying the evolution, taxonomy, genetic characteristics of photosynthesis and organelle biology of the plant. This study fills the gap in the data of the complete chloroplast genome of the edible and ornamental dual-utilization species of roselle. The systematic analysis provided important information for subsequent functional gene mining and molecular breeding practices of the unique crop.

## Figures and Tables

**Table 1:** Base composition characteristics of different sequence regions (LSC, SSC, IRa, IRb).

Sequence Type Characteristics	Base Type	Number	%
Large-Scale Copy Region (LSC) Feature	A	28,931	32.03%
	C	15,998	17.71%
	G	15,132	16.75%
	T	30,266	33.51%
	GC	31,130	34.46%
	All	90,327	100.00%
Small-Scale Copy Region (SSC) Feature	A	6603	33.66%
	C	3232	16.48%
	G	2922	14.90%
	T	6860	34.97%
	GC	6154	31.37%
	All	19,617	100.00%
Inverse Repeat Sequence a (IRa) Feature	A	7493	28.55%
	C	5393	20.55%
	G	5802	22.11%
	T	7554	28.79%
	GC	11,195	42.66%
	All	26,242	100.00%
Inverse Repeat Sequence b (IRb) Feature	A	7554	28.79%
	C	5802	22.11%
	G	5393	20.55%
	T	7493	28.55%
	GC	11,195	42.66%
	All	26,242	100.00%

**Table 2:** Gene annotation of the chloroplast genome of *Hibiscus sabdariffa* L.

Category	Gene Group	Gene Name
Photosynthesis	Subunits of photosystem I	*psaA*, *psaB*, *psaC*, *psaI*, *psaJ*
	Subunits of photosystem II	*psbA*, *psbB*, *psbC*, *psbD*, *psbE*, *psbF*, *psbH*, *psbI*, *psbJ*, *psbK*, *psbL*, *psbM*, *psbN*, *psbT*, *psbZ*
	Subunits of NADH dehydrogenase	*ndhA* *, *ndhB* *(2), *ndhC*, *ndhD*, *ndhE*, *ndhF*, *ndhG*, *ndhH*, *ndhI*, *ndhJ*, *ndhK*
	Subunits of the cytochrome b/f complex	*petA*, *petB* *, *petD* *, *petG*, *petL*, *petN*
	Subunits of ATP synthase	*atpA*, *atpB*, *atpE*, *atpF* *, *atpH*, *atpI*
	Large subunit of Rubisco	*rbcL*
	Subunits of photochlorophyllide reductase	-
Self-replication	Proteins of the large ribosomal subunit	*rpl14*, *rpl16* *, *rpl2* *(2), *rpl20*, *rpl22*, *rpl23* (2), *rpl32*, *rpl33*, *rpl36*
	Proteins of the small ribosomal subunit	*rps11*, *rps12* **(2), *rps14*, *rps15*, *rps16* *, *rps18*, *rps19*, *rps2*, *rps3*, *rps4*, *rps7* (2), *rps8*
	Subunits of RNA polymerase	*rpoA*, *rpoB*, *rpoC1* *, *rpoC2*
	Ribosomal RNAs	*rrn16* (2), *rrn23* (2), *rrn4.5* (2), *rrn5* (2)
	Transfer RNAs	*trnA-UGC* *(2), *trnC-GCA*, *trnD-GUC*, *trnE-UUC*, *trnF-GAA*, *trnG-GCC*, *trnG-UCC* *, *trnH-GUG*, *trnI-CAU* (2), *trnI-GAU* *(2), *trnK-UUU* *, *trnL-CAA* (2), *trnL-UAA* *, *trnL-UAG*, *trnM-CAU*, *trnN-GUU* (2), *trnP-UGG*, *trnQ-UUG*, *trnR-ACG* (2), *trnR-UCU*, *trnS-GCU*, *trnS-GGA*, *trnS-UGA*, *trnT-GGU*, *trnT-UGU*, *trnV-GAC* (2), *trnV-UAC* *, *trnW-CCA*, *trnY-GUA*, *trnfM-CAU*
Other genes	Maturase	*matK*
	Protease	*clpP* **
	Envelope membrane protein	*cemA*
	Acetyl-CoA carboxylase	*accD*
	c-type cytochrome synthesis gene	*ccsA*
	Translation initiation factor	-
	other	-
Genes of unknown function	Conserved hypothetical chloroplast ORF	*ycf1*(2), *ycf2*(2), *ycf3* **, *ycf4*

Note: Gene *: Contains one intron; Gene **: Contains two introns; Gene: Pseudogene; Gene (2): Gene with copy number greater than 1, with copy number indicated in parentheses.

**Table 3:** Relative synonymous codon usage analysis of *Hibiscus sabdariffa* L.

Symbol	Codon	No.	RSCU	Symbol	Codon	No.	RSCU	Symbol	Codon	No.	RSCU
Ter *	UAA	43	1.6539	Arg	AGA	406	1.7862	Lys	AAA	893	1.5174
Ter *	UAG	18	0.6924	Arg	AGG	146	0.642	Lys	AAG	284	0.4826
Ter *	UGA	17	0.6537	Arg	CGA	315	1.3854	Leu	CUA	316	0.7896
Ala	GCA	340	1.046	Arg	CGC	108	0.4752	Leu	CUC	152	0.3798
Ala	GCC	213	0.6552	Arg	CGG	93	0.4092	Leu	CUG	154	0.3846
Ala	GCG	169	0.52	Arg	CGU	296	1.302	Leu	CUU	495	1.2366
Ala	GCU	578	1.7784	Ser	AGC	101	0.3588	Leu	UUA	794	1.9836
Cys	UGC	58	0.4604	Ser	AGU	343	1.2192	Leu	UUG	491	1.2264
Cys	UGU	194	1.5396	Ser	UCA	359	1.2762	Met	AUA	0	0
Asp	GAC	184	0.4058	Ser	UCC	259	0.9204	Met	AUC	0	0
Asp	GAU	723	1.5942	Ser	UCG	143	0.5082	Met	AUG	531	7
Glu	GAA	897	1.5012	Ser	UCU	483	1.7166	Met	AUU	0	0
Glu	GAG	298	0.4988	Thr	ACA	351	1.2072	Met	CUG	0	0
Phe	UUC	429	0.6646	Thr	ACC	228	0.784	Met	GUG	0	0
Phe	UUU	862	1.3354	Thr	ACG	136	0.4676	Met	UUG	0	0
Gly	GGA	622	1.5452	Thr	ACU	448	1.5408	Asn	AAC	255	0.467
Gly	GGC	177	0.4396	Val	GUA	476	1.5244	Asn	AAU	837	1.533
Gly	GGG	288	0.7156	Val	GUC	149	0.4772	Pro	CCA	265	1.1276
Gly	GGU	523	1.2992	Val	GUG	168	0.538	Pro	CCC	180	0.766
His	CAC	140	0.5036	Val	GUU	456	1.4604	Pro	CCG	129	0.5488
His	CAU	416	1.4964	Trp	UGG	399	1	Pro	CCU	366	1.5576
Ile	AUA	622	0.9468	Tyr	UAC	171	0.3972	Gln	CAA	632	1.551
Ile	AUC	370	0.5631	Tyr	UAU	690	1.6028	Gln	CAG	183	0.449
Ile	AUU	979	1.4901								

Note: Symbol: Three-letter amino acid abbreviation, ‘*’ denotes stop codon; Codon: Codon; No.: Number of codons; RSCU: Codon preference.

**Table 4:** Analysis of scattered sequence repeats in the chloroplast genome of *Hibiscus sabdariffa* L.

Length	F	P	R	C	Total
30	10	7	6	1	24
31	6	4	2	0	12
32	4	0	3	1	8
33	2	1	2	1	6
34	4	4	1	0	9
35	0	0	1	0	1
36	1	1	0	0	2
38	2	1	0	0	3
39	1	0	1	0	2
40	1	2	0	0	3
41	2	0	0	0	2
46	0	1	0	0	1
52	1	0	0	0	1
59	0	0	1	0	1
26,242	0	1	0	0	1
Total	34	22	17	3	76

Note: Length represents the length of the repetitive sequence; F denotes forward repeats, P denotes palindromic repeats, R denotes reverse repeats, and C denotes complementary repeats; Total represents the number of all repeats.

## Data Availability

The original contributions presented in this study are included in the article. Further inquiries can be directed to the corresponding authors. All original data (including sequencing reads and annotated genomes) supporting the reported results have been submitted to NCBI GenBank (Submission ID: PX363576).
